# MANAGEMENT OF NONCOGNITIVE NEUROPSYCHIATRIC SYMPTOMS OF DEMENTIA IN NURSING HOME RESIDENTS

**DOI:** 10.1093/geroni/igz038.1874

**Published:** 2019-11-08

**Authors:** Prasanna Basnet, Gayle Acton, Jane D Champion

**Affiliations:** The University of Texas at Austin, Austin, Texas, United States

## Abstract

Background: It is challenging for nursing home (NH) staff to manage non-cognitive neuropsychiatric symptoms (NPS) of dementia. There is a need for an assessment of staff knowledge regarding non-pharmacological approaches to manage NPS of dementia. This assessment will inform development of policies/procedures to assist NH staff for management of problematic behaviors in residents with dementia (RWD) using non-pharmacological approaches thereby complying with CMS directives to reduce psychotropic medication use. Methods: NH staff members were interviewed using semi-structured interview methods. Interviews continued until thematic saturation was reached. A total of 17 interviews were completed. Findings: Forgetfulness, hallucinations, anger, agitation, combativeness are the most common problematic dementia behaviors. Participants reported that these behaviors make activities of daily living (ADL) care challenging and time-consuming. Redirection, distraction, and recreational activities are the most common approaches identified by participants to manage non-cognitive NPS using non-pharmacological techniques. Participants reported rushing residents to get things done hinders cooperation and escalate problematic behavior. Some participants believed the use of a low dose PRN benzodiazepine is effective as this calms the resident with dementia and reduces NH staff time requirements for assistance with ADL care. Participants described adverse reactions such as weight loss, and general decline as an outcome of somnolence secondary to psychotropic medications use. Implications: NH staff who participated in this project had not received any formal education or instruction concerning non-pharmacological approaches for the management of non-cognitive NPS. NH protocols are indicated for non-pharmacologic behavior management techniques prior to the administration of psychotropic medications.

